# Steroid-dependent metabolic rewiring reveals novel therapeutic and imaging approaches for glioblastoma

**DOI:** 10.1126/sciadv.adx6539

**Published:** 2026-01-23

**Authors:** Maria Francesca Allega, Ruhi Deshmukh, Theresa Hillinger, Alena Akhmetshina, Anaïs Oudin, Robert Bielik, Dmitry Soloviev, Victor H. Villar, Tobias Ackermann, Guillaume Bourmeau, Sandeep K. Chahal, Katrina H. Stevenson, Colin Nixon, Robin Shaw, Gillian M. Morrison, Anthony J. Chalmers, Steven M. Pollard, Morten Lund-Johansen, Rolf Bjerkvig, Giorgio Seano, Simone P. Niclou, Einar O. Vik-Mo, David Y. Lewis, David Sumpton, Saverio Tardito

**Affiliations:** ^1^ Cancer Research UK Scotland Institute, Garscube Estate, Switchback Road, Glasgow, G61 1BD, UK.; ^2^ Medical University of Vienna, Comprehensive Cancer Center, Center for Cancer Research, Vienna 1090, Austria.; ^3^ NORLUX Neuro-Oncology Laboratory, Department of Cancer Research, Luxembourg Institute of Health, L-1210 Luxembourg, Luxembourg.; ^4^ Animal Facility, Department of Cancer Research, Luxembourg Institute of Health, L-4354 Luxembourg, Luxembourg.; ^5^ Institut Curie, INSERM U1021, CNRS UMR3347, Paris-Saclay University, 91400 Orsay, France.; ^6^ School of Cancer Sciences, University of Glasgow, Glasgow, G61 1QH, UK.; ^7^ Cancer Research UK Scotland Centre and Centre for Regenerative Medicine, Institute for Regeneration and Repair, University of Edinburgh, Edinburgh, EH16 4UU, UK.; ^8^ Department of Neurosurgery, Haukeland University Hospital, Bergen, Norway.; ^9^ Department of Clinical Medicine, University of Bergen, Bergen, Norway.; ^10^ Department of Biomedicine, University of Bergen, Bergen, Norway.; ^11^ Department of Neurosurgery, Qilu Hospital of Shandong University and Brain Science Research Institute Shandong University, Key Laboratory of Brain Functional Remodeling, Shandong, China.; ^12^ Faculty of Science, Technology and Medicine, University of Luxembourg, L-4365 Esch-sur-Alzette, Luxembourg.; ^13^ Vilhelm Magnus Laboratory, Institute for Surgical Research and Department of Neurosurgery and Oslo University Hospital, Oslo, Norway.; ^14^ Institute for Clinical Medicine, Faculty of Medicine, University of Oslo, Oslo, Norway.

## Abstract

Steroid anti-inflammatory drugs, such as dexamethasone, are routinely used to manage brain tumor–associated edema, yet their impact on brain tumor metabolism remains understudied. Here, a metabolomic screen in naïve glioblastoma cells treated with dexamethasone revealed the accumulation of *N*^1^-methylnicotinamide, a nicotinamide *N*-methyltransferase (NNMT) product, through glucocorticoid receptor activation. Using stable isotope-assisted metabolomics in patients with glioblastoma, we showed that nicotinamide conversion into *N*^1^-methylnicotinamide exceeds that into NAD^+^, leading to a ~7-fold accumulation of *N*^1^-methylnicotinamide in tumor compared to surrounding brain tissue. In orthotopic models, NNMT activity was enhanced by dexamethasone selectively in glioblastoma tumors but not in contralateral brain. Leveraging the tumor-specific activity of NNMT, we developed a novel ^11^C-nicotinamide–based positron emission tomography (PET) approach to visualizing glioblastoma tumors. Furthermore, our findings demonstrate that the dexamethasone-induced methionine-dependent nicotinamide methylation becomes detrimental for glioblastoma when combined with a methionine-restricted diet. These results show that steroids rewire methionine and nicotinamide metabolism, enabling the development of innovative PET imaging and metabolic therapies for glioblastoma.

## Teaser

Dexamethasone reprograms glioblastoma nicotinamide metabolism, sensitizing tumors to methionine restriction and enabling PET.

## INTRODUCTION

Malignant gliomas represent ~70% of new cases of malignant primary brain tumors, and glioblastoma is the most aggressive and common malignant glioma in adults. The median survival for patients with glioblastoma is 12 to 15 months, making it one of the deadliest cancer types (*1*). The current World Health Organization classification of brain tumors restricts the diagnosis of glioblastoma to tumors that are wild type for the metabolic enzymes isocitrate dehydrogenase 1 (*IDH1*) and 2 (*IDH2*). Hence, the lack of a genetic mutation defining the metabolic makeup of glioblastomas challenges the design of clinically relevant therapies to effectively target the metabolism of these tumors (*2*). Moreover, the risk factors for glioblastoma are largely unknown, fundamentally impairing impactful preventive interventions and emphasizing the urgent need for more effective diagnostics and therapies.

For the past 60 years (*3*), patients with glioblastoma, before proceeding with surgical resection to debulk the tumor mass, have been ordinarily administered synthetic steroids. These potent anti-inflammatory drugs decrease tumor-associated edema and ameliorate its life-changing symptoms (*4*). Steroids may need to be continued after surgery to reduce perisurgical inflammation and then alongside radio- and chemotherapy to manage treatment-related edema. Among other steroids such as hydrocortisone, cortisone, and prednisolone, dexamethasone represents the drug of choice for patients with glioblastoma due to its long half-life and limited mineralocorticoid activity (*5*). Dexamethasone is commonly administered in the range of 4 to 16 mg/day and up to 100 mg/day in patients with severe symptoms (*6*). Natural corticosteroids are hormones whose synthesis and secretion by the adrenal glands are regulated through the hypothalamic-pituitary-adrenal axis following circadian and ultradian rhythms (*7*). Synthetic glucocorticoids such as dexamethasone are more potent and have a higher bioavailability than their natural counterparts, but both physiological and synthetic glucocorticoids exert most of their functions through their binding target, the glucocorticoid receptor (GR), a nuclear receptor encoded by the *NR3C1* gene (*8*).

Several studies have investigated the combinatorial effects of chemotherapeutic drugs and glucocorticoids, showing that dexamethasone can either protect from or enhance the cytotoxic effects of chemotherapy on glioma cells (*9*–*11*). Glucocorticoids modulate tissue-specific metabolic pathways, particularly glucose and lipid metabolism. They promote gluconeogenesis in the liver (*12*), whereas in skeletal muscle and white adipose tissue, they cause a decrease in glucose uptake antagonizing insulin signaling (*13*) and inducing hyperlipidemia (*14*). In patients with glioblastoma, high-dose dexamethasone (≥8 mg) is associated with higher fasting blood glucose concentrations on postoperative days and increased risk of postoperative complications (*15*). In line with these observations, it has been reported that glucocorticoids compromise survival in three independent cohorts of patients with glioblastoma (*16*). In this study, we interrogated a panel of glioblastoma cell lines cultured in physiologically relevant conditions to identify metabolic reactions consistently altered by clinically relevant doses of dexamethasone. Untargeted metabolomic and transcriptomic analyses revealed that *N*^1^-methylnicotinamide and its synthesizing enzyme nicotinamide *N*-methyltransferase (NNMT) accumulated upon dexamethasone treatment. This effect, conserved in vivo in orthotopic mouse models, together with the differential levels of *N*^1^-methylnicotinamide between tumors and surrounding brain tissue observed in patients with glioblastoma, led us to develop approaches to visualizing glioblastoma with radiometabolic positron emission tomography (PET) imaging and to inhibit tumor progression with a dietary intervention.

## RESULTS

### Dexamethasone elicits a transcriptional signature conserved across naïve glioblastoma cell lines

**Fig. 1.**
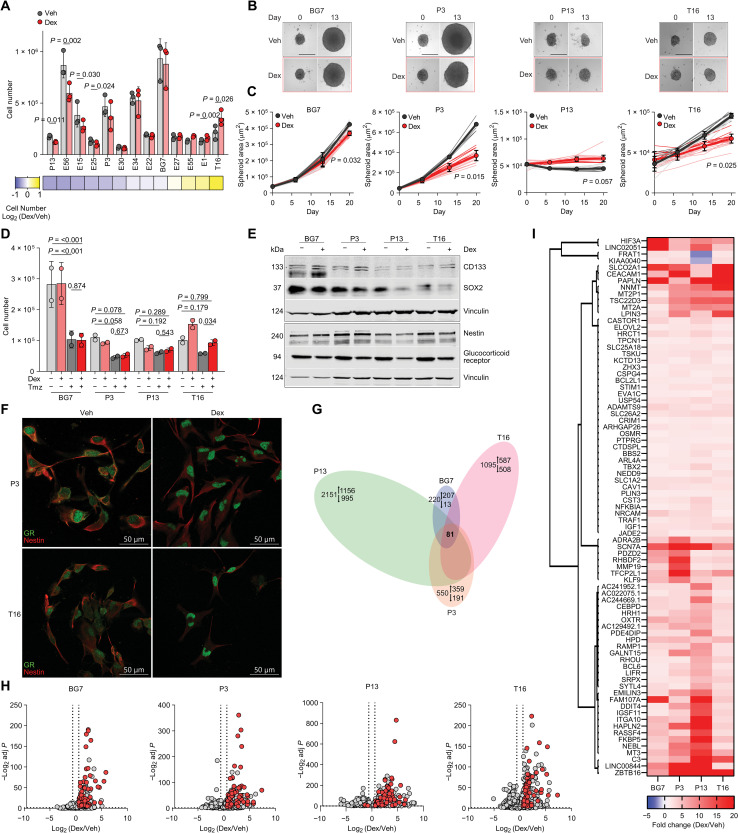
Dexamethasone (Dex) effects on proliferation and transcriptome of glioblastoma cells. (**A**) Number of glioblastoma cells in 2D treated with Veh [(vehicle), ethanol 0.01%] or Dex, 0.1 μM for 9 days. Cell lines are ranked on the effect of Dex. (**B** and **C**) Spheroid growth of cells cultured with Veh or 0.1 μM Dex. (B) Representative images. Scale bars, 400 μm. (C) Thin lines: area of individual spheroids, thicker lines: mean ± SD of six to nine spheroids’ area. (**D**) Number of glioblastoma cells in 2D treated with Veh, 0.1 μM Dex, or 250 µM TMZ for 4 days. (**E**) Western blot of Nestin, CD133, SOX2, and GR in cells cultured in 2D and treated with Veh or 0.1 μM Dex for 3 days. Vinculin, loading control. (**F**) Confocal images of GR and Nestin in cells treated with Veh or 1 μM Dex for 3 days. (**G** to **I**) RNA-seq: (G) number of genes significantly regulated in cells cultured in 2D and treated as in (E). Arrows pointing up- and down-ward indicate the number of genes up- and down-regulated, respectively. Volcano plots (H) highlighting with red points the 81 genes significantly regulated in all four cell lines. Gray points: all other genes. (I) Heatmap of the 81 genes highlighted in (H). Mean ± SD, *n*_exp_ = 3 [(A) and (G) to (I)] and *n*_exp_ = 2 [(C) and (D)]. *P* values are from two-tailed, homoschedastic, Student’s *t* tests: ratio paired (A) or unpaired [(C) and (D)]. In (D), the *P* values refer to the comparison at 20 days. [(G) to (I)] Statistical analysis described in the “RNA sequencing” section.

Despite different clinical practices and the interindividual heterogeneity of their tumors, most patients with glioblastoma receive dexamethasone at some stage of their disease trajectory (*17*). To study the effect of clinically relevant doses of dexamethasone, we treated a panel of 13 naïve glioblastoma cell lines, cultured in serum-free physiologically relevant conditions, with 0.1 μM dexamethasone, a concentration observed in the blood of patients undergoing steroid therapy (*18*, *19*). When tested in two-dimensional (2D) monolayers, the drug had significant antiproliferative effects in four cell lines, no significant effects in seven, and significantly accelerated proliferation in two (Fig. 1A). Four cell lines (BG7, P3, P13, and T16) were selected to represent these three groups of biological responses and were cultured as three-dimensional (3D) spheroids. Notably, the positive effect of dexamethasone on the proliferation of T16 cells was reversed when these cells were grown as 3D spheroids (Fig. 1, B and C). These results show that the effect of dexamethasone on glioblastoma cell proliferation is cell line specific and dependent on the culture conditions. Dexamethasone is often administered to patients with glioblastoma undergoing temozolomide (TMZ) chemotherapy, and it has been shown that the combination associates with poorer prognosis (*20*). As observed in the clinical setting (*21*), the expression of O^6^-methylguanine-DNA methyltransferase (*MGMT*) antagonizes the therapeutic effect of TMZ in glioblastoma cells (fig. S1, A and B). In general, dexamethasone did not alter TMZ response, except in MGMT-positive T16 cells cultured in 2D (Fig. 1D and fig. S1C). Similarly, dexamethasone did not consistently alter the expression of the stemness markers Nestin, CD133, and SOX2, which are involved in maintaining a stem-like state (Fig. 1E). In contrast, dexamethasone consistently down-regulated GR expression in all cell lines (Fig. 1E). This observation, explained by the presence of glucocorticoid repressive elements in the promoter of the GR gene (*NR3C1*) (*22*), suggests that in glioblastoma cells, the phenotypic effects of dexamethasone are mediated by activation of its canonical target, GR. Immunofluorescence staining of the GR shows its nuclear translocation upon dexamethasone treatment demonstrating receptor functionality in glioblastoma cells (Fig. 1F). RNA sequencing (RNA-seq) of four glioblastoma cell lines treated with dexamethasone revealed that fewer than 3% of the 2792 genes regulated in at least one cell line, were consistently regulated across all four, indicating a cell line–specific transcriptional response to the drug (Fig. 1, G to I).

### Dexamethasone reprograms glioblastoma cells toward an astrocytic metabolism

**Fig. 2.**
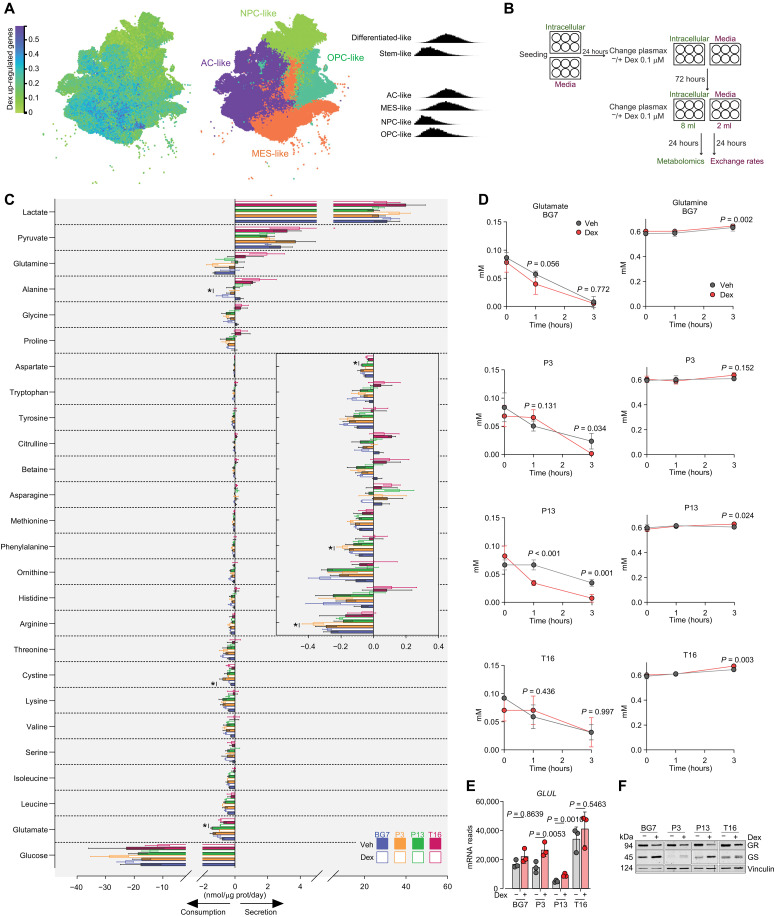
Dex reprograms glioblastoma cells toward an astrocytic metabolism. (**A**) Dex signature corresponding to the 79 genes significantly up-regulated in all four cell lines, overlaid to the harmonized dataset for glioblastoma. Left: In blue, cells expressing high levels of the Dex signature genes. Middle: Classification of neoplastic cells according to the Neftel *et al.* (*62*) cell states in the harmonized dataset. Right: Ridgeline plots of the expression of dex up-regulated genes in Neftel *et al.* (*62*) cell states and on a differentiation gradient. NPC, neural progenitor; OPC, oligodendrocyte progenitor; AC, astrocyte; MES, mesenchymal. (**B**) Schematic of the experiments presented in (C). (**C**) Exchange rates of metabolites between cells and media calculated as described in Materials and Methods. The inset shows the consumption/secretion of the metabolites (main *y*-axis labels) with a narrower *x*-axis values range. Bars represent mean ± SD, *n*_exp_ = 3. * represents *P* < 0.05 obtained with a two-tailed, Student’s *t* tests for paired samples. (**D**) Glutamate and glutamine concentrations were measured in spent culture media with a YSI biochemistry analyzer. Cells were pretreated for 72 hours with Veh or 0.1 μM Dex, and then the respective media were refreshed and sampled at the indicated times. *n*_exp_ = 3. *P* values refer to a two-tailed, homoscedastic Student’s *t* tests for unpaired samples comparing Veh to Dex. (**E**) Expression levels of glutamine synthetase (*GLUL*) mRNA obtained by RNA-seq. *n*_exp_ = 3 as indicated by the data points. Bars represent mean ± SD. *P* values were calculated with DESeq2 using a Benjamini-Hochberg method to correct for multiple testing. (**F**) Western blot analysis showing the expression of glutamine synthetase (GS) and GR in four glioblastoma cell lines cultured as monolayers and treated with vehicle or 0.1 μM Dex for 3 days. Vinculin is shown as a loading control. prot, protein.

A manually curated gene set enrichment analysis (GSEA) for glioblastoma-specific cell states revealed that dexamethasone reprograms glioblastoma cells toward a mesenchymal- and astrocytic-like state (fig. S2, A and B). Consistent with this, overlay of the dexamethasone-driven signature with a harmonized single-cell database for glioblastoma landscapes (*23*) shows that the gene signature is preferentially expressed in glioblastoma cells with a mesenchymal- and astrocytic-like profile (Fig. 2A). Among these genes, we found *SLC1A2* and *SLC25A18*, encoding two astrocytic solute carriers that transport glutamate across the plasma-membrane and inner mitochondrial membrane, respectively (fig. S2C).

To profile the metabolic effects of dexamethasone in glioblastoma cells, we measured consumption and secretion rates of 26 metabolites present in the physiological medium Plasmax (Fig. 2, B and C). Dexamethasone modulated the exchange rate of several nutrients but rather inconsistently across the four lines. Notably, glutamate was, on average, the second most avidly consumed nutrient across the cell lines (Fig. 2C), in line with the expression of the high-affinity glutamate carrier SLC1A2 (fig. S2C). Other anaplerotic nutrients were either not consistently consumed (i.e., glutamine; Fig. 2C) or even secreted in the medium by all cell lines (i.e., pyruvate; Fig. 2C). Since in this assay the glutamate available in the medium was cleared by all cell lines, likely underestimating its consumption, the glutamate concentrations were measured in spent media at earlier time points (Fig. 2D). Within 3 hours, glutamate was depleted by all cell lines with a rate that was accelerated by dexamethasone in the three cell lines with the highest expression of SLC1A2 (fig. S2C). Conversely, all cell lines secreted glutamine over time (Fig. 2D). A net secretion of glutamine is necessarily underpinned by the biosynthetic activity of glutamine synthetase (GS), another astrocytic marker whose gene (*GLUL*) and protein expression were enhanced by dexamethasone treatment (Fig. 2, E and F). Overall, these results reveal that glutamate is the preferential amino acid consumed by glioblastoma cells and is used, at least in part, to produce glutamine via glutamine synthetase, two key features of astrocytic metabolism enhanced by dexamethasone in glioblastoma cells.

### Untargeted metabolomics reveals NNMT-dependent *N*^1^-methylnicotinamide accumulation in dexamethasone-treated cells

**Fig. 3.**
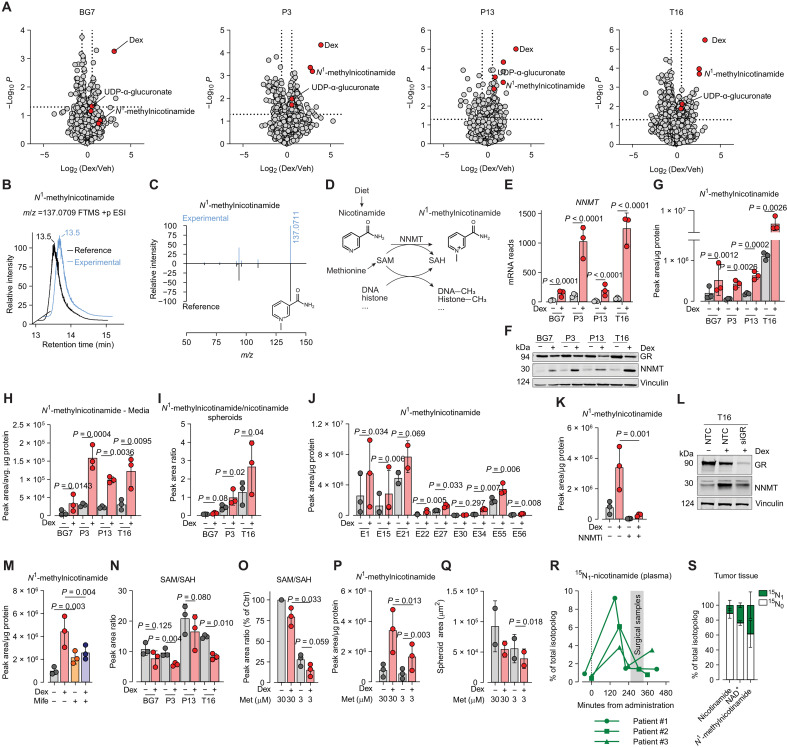
*N*^1^-methylnicotinamide accumulates in Dex-treated cells. (**A**) Volcano plots from untargeted metabolomics of glioblastoma cells treated for 4 days with Veh or 0.1 μM Dex (as in Fig. 2B). Red points: metabolites significantly regulated by Dex in ≥3 out of 4 cell lines. Gray points: all other metabolites. (**B**) Overlaid extracted ion chromatograms from T16 cells and *N*^1^-methylnicotinamide chemical reference. (**C**) Mirror tandem mass spectrometry spectra comparing parent ion [mass/charge ratio (*m/z*) 137.0711] and fragments from T16 extracts and *N*^1^-methylnicotinamide reference. (**D**) NNMT-catalyzed reaction. (**E**) NNMT mRNA levels by RNA-seq after 3 days of Dex treatment. (**F**) Western blot of NNMT and GR in four glioblastoma lines treated with Veh or Dex (0.1 μM, 3 days). Vinculin, loading control. (**G** and **H**) Intracellular (G) and extracellular (H) *N*^1^-methylnicotinamide levels of cells incubated for 4 days with Veh or 0.1 μM Dex. (**I**) *N*^1^-methylnicotinamide/nicotinamide ratio in spheroids incubated for 4 days with Veh or 0.1 μM Dex. (**J**) Intracellular *N*^1^-methylnicotinamide levels in cells incubated as in (G). (**K**) *N*^1^-methylnicotinamide levels in T16 cells incubated as in (G) with Veh or 10 μM NNMT inhibitor (NNMTi, JBSNF-000088). (**L**) Western blot of NNMT and GR in T16 cells transfected with GR siRNAs or nontargeting control (NTC) for 4 days and then treated with 0.1 μM Dex for 2 days. Vinculin, loading control. (**M**) Intracellular *N*^1^-methylnicotinamide levels in T16 cells incubated as in (G) ± 5 μM mifepristone (Mife). (**N**) SAM/SAH ratio in cells incubated as in (G). (**O** and **P**) Levels of SAM/SAH (O) and *N*^1^-methylnicotinamide (P) in T16 cells incubated as in (G) with the indicated concentrations of methionine (Met). SAM/SAH ratio expressed as the percentage of control (no Dex, 30 μM Met). (**Q**) Spheroid growth of T16 cells cultured for 27 days with Veh or 0.1 μM Dex and indicated methionine concentrations. (**R**) Plasma levels of ^15^N_1_-labeled nicotinamide in patients with glioblastoma (*n* = 3) sampled following oral administration of ^15^N_1_ nicotinamide (time 0). Surgical sampling time window is indicated with a gray box. (**S**) Fractional enrichment of ^15^N-labeled metabolites in matched tumor tissue from patients described in (R). Mean ± SD, *n*_exp_ = 3 for all experiments unless otherwise noted [(J): E21 and E30 *n*_exp_ = 2; (Q): *n*_exp_ = 2)]. *P* values are from two-tailed, homoschedastic, Student’s *t* tests: ratio paired [(G) to (K) and (M) to (Q)] or unpaired (A). (E) *P* values were calculated with DESeq2 using a Benjamini-Hochberg method to correct for multiple testing. FTMS, Fourier transform mass spectrometry; +p, positive polarity; ESI, electrospray ionization.

To identify further metabolic alterations consistently driven by dexamethasone, we performed untargeted metabolomics on intracellular extracts and identified five features whose levels were significantly different in at least three of the four glioblastoma cell lines treated with dexamethasone (Fig. 3A). Among these features, dexamethasone, UDP-α-glucuronic acid and *N*^1^-methylnicotinamide were consistently more abundant in all dexamethasone-treated cell lines as compared to controls (Fig. 3A). Comparison of retention times and fragmentation patterns of the ions detected in the experimental samples with those of chemical standards confirmed the identity of *N*^1^-methylnicotinamide and UDP-α-glucuronate (Fig. 3, B and C, and fig. S3, A and B).

The mRNA and protein expression of UDP-glucose dehydrogenase (UGDH), which catalyzes the production of UDP-α-glucuronic acid, were not affected by dexamethasone treatment (fig. S3, C and D). Conversely, *NNMT*, encoding for the enzyme responsible for the synthesis of *N*^1^-methylnicotinamide (Fig. 3D), was among the 79 transcripts up-regulated by dexamethasone in all four glioblastoma cell lines (Figs. 1I and 3E), and its expression pattern was confirmed at the protein level (Fig. 3F). Targeted metabolic analysis showed that *N*^1^-methylnicotinamide levels were consistently increased by dexamethasone in glioblastoma cell monolayers (Fig. 3G), in their spent media (Fig. 3H), and in 3D spheroids (Fig. 3I). Levels of *N*^1^-methylnicotinamide were measured in a further nine patient-derived glioblastoma lines, seven of which showed a significant accumulation of the metabolite when treated with dexamethasone (Fig. 3J). These results demonstrate that dexamethasone-dependent increases in NNMT and *N*^1^-methylnicotinamide are widely conserved in different culture conditions across heterogeneous glioblastoma cell lines.

The causality link between NNMT expression and *N*^1^-methylnicotinamide levels was demonstrated by the effects of JBSNF-000088, an NNMT inhibitor, which completely prevented accumulation of *N*^1^-methylnicotinamide in dexamethasone-treated T16 cells (Fig. 3K), without affecting their growth as spheroids (fig. S3E).

Silencing GR in T16 cells blunted dexamethasone-induced NNMT up-regulation, demonstrating its GR dependence (Fig. 3L). To validate this with an orthogonal pharmacologic approach, we used mifepristone, a GR antagonist with partial agonist activity (*24*). Due to its partial agonist activity, mifepristone increased *N*^1^-methylnicotinamide levels when used alone, but blocked its accumulation in dexamethasone-treated cells (Fig. 3M). Together, these results demonstrate that NNMT is a previously unrecognized transcriptional target of the GR in glioblastoma cells.

*N*^1^-methylnicotinamide is synthesized by NNMT from nicotinamide and *S*-adenosylmethionine (SAM), a cosubstrate of several methyltransferases (Fig. 3D). Consistent with an increased flux of methyl groups toward *N*^1^-methylnicotinamide, dexamethasone decreased the ratio between SAM and *S*-adenosylhomocysteine (SAH), a proxy for the methylation capacity of the cell (Fig. 3N). Nevertheless, dexamethasone treatment did not affect global levels of DNA methylation or histone 3 methylation status (fig. S3, F and G). The availability of activated methyl groups for methylation reactions largely depends on methionine concentrations. When T16 glioblastoma cells were challenged with subphysiological levels of methionine (3 μM), their SAM/SAH ratio dropped by 75% (Fig. 3O). Notably, under these methionine-restricted conditions, dexamethasone further depleted the SAM/SAH ratio while significantly increasing *N*^1^-methylnicotinamide levels (Fig. 3, O and P). These results demonstrate an unreported (*25*) high affinity of NNMT for SAM in living cells and link reduced methylation capacity to the enhanced antiproliferative effect of dexamethasone observed upon methionine restriction (Fig. 3Q).

### Stable isotope-assisted metabolic tracing in patients with glioblastoma reveals a prominent role of NNMT

To test the relevance of NNMT activity for the metabolism of human tumors, ^15^N-labeled nicotinamide was orally administered to three patients with glioblastoma before undergoing tumor debulking surgery. At the time when the surgical samples were collected, ~1.5% of the circulating nicotinamide pool was labeled (Fig. 3R). In the resected tumor tissue, ^15^N-nicotinamide was 15% of the total pool, revealing the efficient glioblastoma uptake of diet-derived nicotinamide from systemic circulation (Fig. 3S). The fraction of ^15^N-labeled *N*^1^-methylnicotinamide was ~40% of the total pool, showing that NNMT activity in the tumor tissue leads to *N*^1^-methylnicotinamide accumulation. Nicotinamide is also a substrate of nicotinamide phosphoribosyltransferase (NAMPT) that converts it to nicotinamide adenine dinucleotide (NAD^+^), whose ^15^N-labeled fraction accounted for ~24% of the total pool in the tumor tissue (Fig. 3S). This isotope distribution indicates that in human glioblastoma, the NNMT-dependent conversion of nicotinamide to *N*^1^-methylnicotinamide exceeds that of nicotinamide to NAD^+^.

### Dexamethasone impairs glioblastoma tumor growth and boosts tumor-specific *N*^1^-methylnicotinamide production

**Fig. 4.**
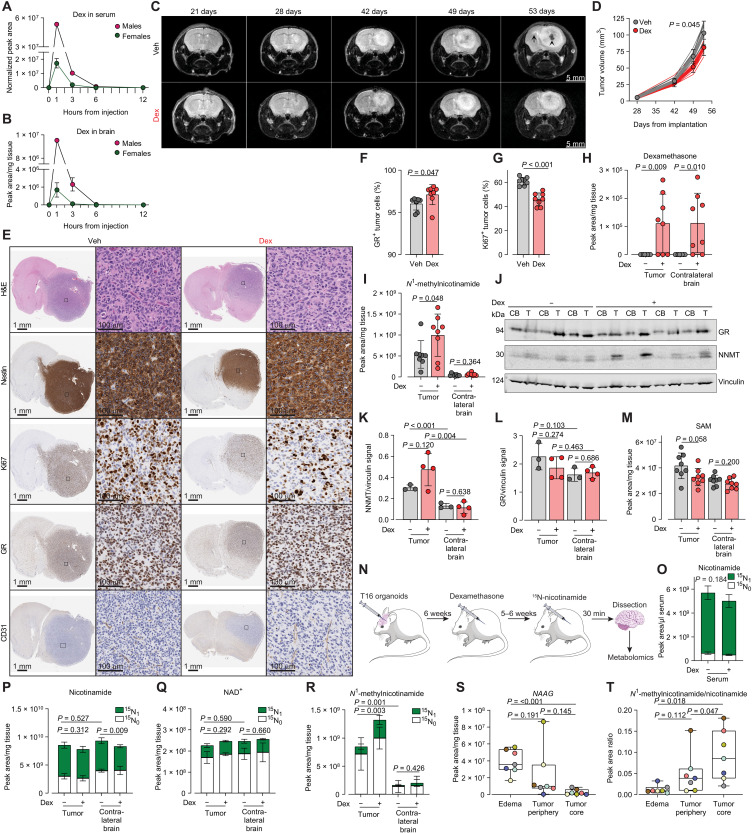
Dex boosts tumor-specific *N*^1^-methylnicotinamide production. (**A** and **B**) Dexamethasone (Dex) levels in serum (A) and brain tissue (B) of tumor-free NSG mice intraperitoneally injected with dexamethasone-21-phosphate (2 mg/kg). Mean ± SD, *n*_mice_ = 2. (**C**) Representative brain T2 MRI images of female nude mice transplanted with T16 organoids and treated five times/week with dexamethasone-21-phosphate (2 mg/kg) from 3 weeks posttransplantation. Tumor contralateral infiltration (arrow) and necrosis (arrowhead) are visible. (**D**) Tumor volumes from MRI of mice described in (C). Thin lines: individual tumors, thick lines mean ± SD, *n*_mice_ = 8. (**E**) Hematoxylin and eosin (H&E) and immunohistochemistry (IHC) of consecutive brain sections from mice described in (C) sampled 53 days postimplantation. Right: higher magnifications of squares in the left. (**F** and **G**) Percentage of GR- and Ki67-positive tumor cells quantified in mice described in (C). Mean ± SD, *n*_mice_ = 8. (**H**) Dex levels in brain tissue of mice described in (C). Mean ± SD, *n*_mice_ = 8. (**I**) *N*^1^-methylnicotinamide levels in the tumor and contralateral brain tissue of mice described in (C). Mean ± SD, *n*_mice_ = 8. (**J**) Western blot analysis of GR and NNMT in tumor (T) and matched contralateral brain (CB) tissue of mice described in (C). Vinculin, loading control. Quantification of NNMT (**K**) and GR (**L**) signal from (J). Mean ± SD, Veh: *n*_mice_ = 3, Dex: *n*_mice_ = 4. (**M**) SAM levels in tumor and contralateral brain tissue of mice described in (C). Mean ± SD, *n*_mice_ = 8. (**N**) Experimental design for ^15^N-nicotinamide tracing in NSG female mice implanted with T16 organoids. (**O** to **R**) ^15^N-nicotinamide [(O) and (P)], ^15^N-NAD^+^ (Q), and ^15^N-N^1^-methylnicotinamide (R) in the tumor and contralateral brain tissue [(P), (Q), and (R)] or serum (O) of Veh and Dex treated mice. Mean ± SD, Veh: *n*_mice_ = 5, Dex: *n*_mice_ = 4. (**S** and **T**) Levels of *N*-acetylaspartylglutamate (NAAG) (S) and *N*^1^-methylnicotinamide/nicotinamide (T) in stereotactically collected samples from patients with glioblastoma (edema, tumor periphery, and tumor core). Individual patients are color-coded. *n*_patients_ = 7. Boxes represent the 25th to 75th percentiles, medians are shown by lines, and whiskers indicate minimum-maximum values. *P* value in (D) was assessed by CGGC permutation test (1000 permutations). All other *P* values were calculated using two-tailed, homoscedastic Student’s *t* tests: ratio paired [(S) and (T)] or unpaired [(F) to (M) and (O) to (R)]. In (O) to (R), levels of ^15^N_1_ metabolites were compared.

To study the effect of dexamethasone in vivo, mice were administered dexamethasone-21-phosphate (herein dexamethasone, Dex), a water soluble prodrug (*26*) that resulted in detectable levels of dexamethasone in both blood circulation and brain tissue for 3 to 6 hours after administration (Fig. 4, A and B). The effects of dexamethasone on glioblastoma were tested in immunodeficient female mice orthotopically implanted with female patient-derived T16 organoids, a metabolically characterized model (*27*) whose progression could be followed noninvasively by magnetic resonance imaging (MRI) (*28*). Tumor progression of vehicle- and dexamethasone-treated mice was monitored over time by MRI, showing hallmarks of human glioblastoma tumors, such as brain parenchyma infiltration and intratumoral necrosis (Fig. 4C). Quantification of tumor volumes over time showed a significant growth delay in tumors treated with dexamethasone (Fig. 4D). Histological analysis of these tumors showed homogeneous expression of the human neural stem cell marker Nestin, infiltration of extravascular CD31^+^ endothelial cells, nuclear localization of GR, and frequent positivity for the proliferative marker Ki67 (Fig. 4E). The percentage of GR-positive cells was significantly increased in dexamethasone-treated tumors (Fig. 4F). Conversely, the percentage of Ki67^+^ cells was significantly decreased by dexamethasone (Fig. 4G), demonstrating that the steroid engages with GR in the tumor and impairs the proliferation of glioblastoma cells, thus recapitulating the results obtained in culture with T16 spheroids (Fig. 1, B and C).

Metabolic analyses of tissues from dexamethasone-treated mice detected comparable dexamethasone levels in tumors and in normal brain tissue from the contralateral hemisphere (Fig. 4H), ruling out concentration-dependent effects and differences in drug uptake between normal brain and tumor. Conversely, levels of *N*^1^-methylnicotinamide were found to be ~10-fold more abundant in the tumor as compared to the contralateral brain (Fig. 4I). Notably, dexamethasone treatment selectively increased by 85% the levels of *N*^1^-methylnicotinamide in the tumor but not in the contralateral brain (Fig. 4I). Consistent with the levels of its product, NNMT expression was also increased by dexamethasone in tumors but not in contralateral normal tissue (Fig. 4, J and K). This difference was not explained by the baseline expression of GR in the two tissues (Fig. 4L). Moreover, dexamethasone treatment decreased the levels of the NNMT substrate SAM, specifically in the tumor tissue (Fig. 4M), recapitulating the results obtained with glioblastoma cells in culture (Fig. 3, N to O).

Next, we used stable isotope-assisted metabolomics to assess NNMT activity in tumor-bearing mice (Fig. 4N). Thirty minutes after administration of ^15^N-nicotinamide, its enrichment reached ~90% in blood circulation, which was comparable between control- and dexamethasone-treated animals (Fig. 4O). Overall, levels of ^15^N-nicotinamide fractional enrichment (Fig. 4P), and its conversion to ^15^N-NAD^+^ (Fig. 4Q) were all comparable between tumor and contralateral brain tissue and unaffected by dexamethasone treatment. In contrast, levels of *N*^1^-methylnicotinamide labeled from nicotinamide were significantly higher in tumors compared to contralateral brains in vehicle-treated mice and selectively increased by dexamethasone treatment in tumors, but not in contralateral brains (Fig. 4R).

To test whether the differential activity of NNMT in glioblastoma and normal brain observed in orthotopic xenografts was conserved in humans, we measured levels of *N*^1^-methylnicotinamide in tissues from patients with glioblastoma stereotactically sampled from the tumor core, the tumor periphery, or the edematous brain tissue surrounding the tumor. Levels of *N*-acetylaspartylglutamate, a neuropeptide more abundant in human brain (*29*) than in tumors (*30*) (*31*), accurately discriminated edematous brain tissue from glioblastoma tumor core (Fig. 4S), metabolically validating the accuracy of the stereotactic sampling. In the same patient samples, *N*^1^-methylnicotinamide levels progressively increased from the periphery to the tumor core, where they reached a mean value ~7-fold higher than in the edematous tissue (Fig. 4T). Overall, these results demonstrate that expression and activity of NNMT are increased in glioblastoma tissue compared to normal brain and that conversion of blood-borne nicotinamide to *N*^1^-methylnicotinamide is selectively enhanced by dexamethasone in the tumor.

### Glioblastoma-specific nicotinamide metabolism is visualized by metabolic PET imaging and sensitizes to methionine-restricted diet

**Fig. 5.**
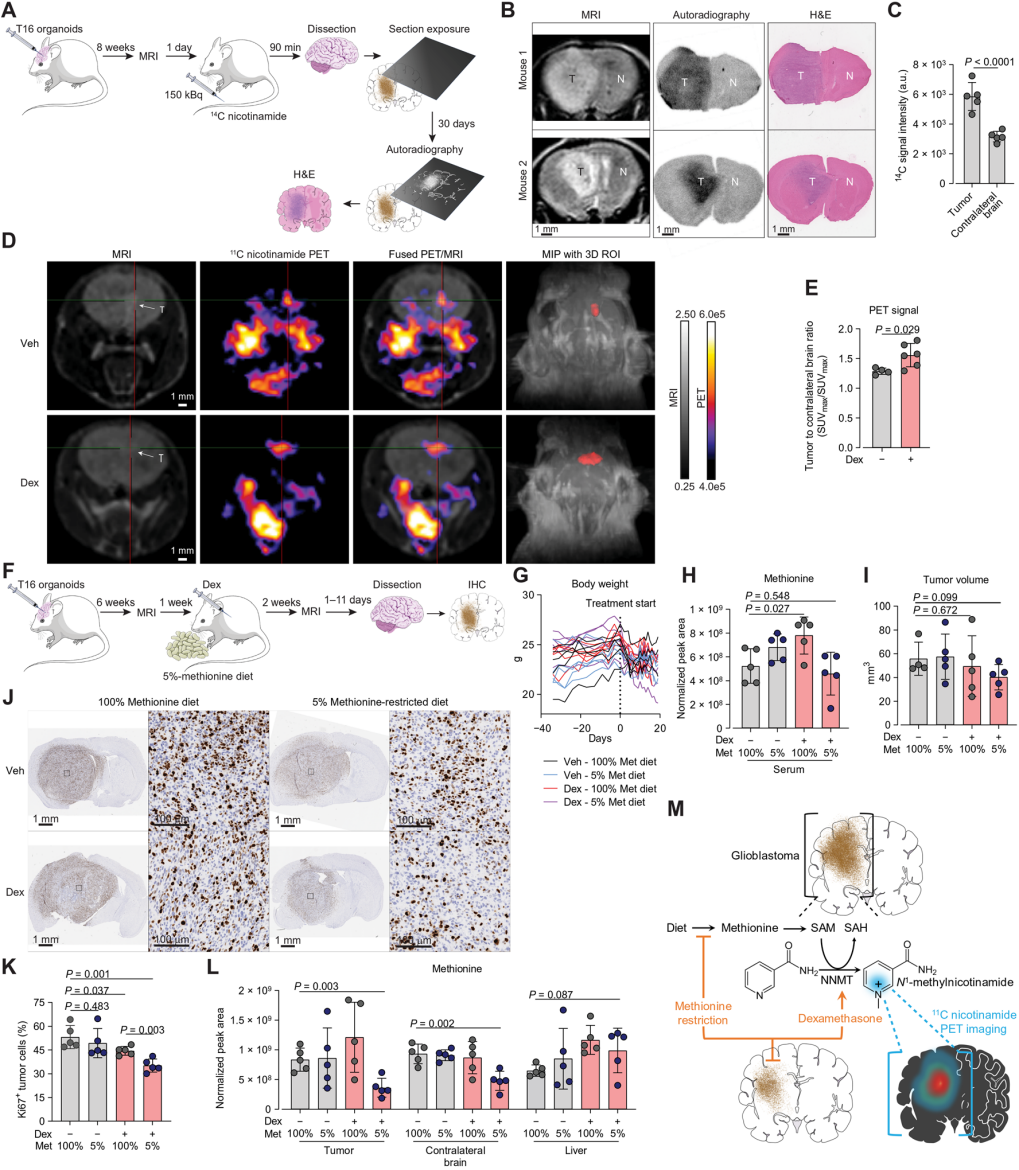
Dex-increased NNMT activity offers imaging and therapeutic approaches. (**A**) Experimental design for brain autoradiography with [carbonyl-^14^C]nicotinamide in NSG female mice bearing T16 glioblastoma tumors. (**B**) Representative MRI, ex vivo autoradiography, and H&E-stained brain sections from mice treated as in (A); autoradiography and H&E were performed on the same sections. (**C**) Quantification of the ^14^C autoradiography signal in tumor (T) or normal brain (N) 90 min after [carbonyl-^14^C]nicotinamide administration. Mean ± SD, *n*_mice_ = 5. (**D**) Representative PET-MRI images of mice with T16 tumors treated with Veh and Dex as described in Materials and Methods. Mice were administered ^11^C nicotinamide, and PET images were acquired 60 min postinjection. Min/max PET signal values are shown in becquerel per milliliter. T2-weighted MRI images were overlayed with PET images, and maximum intensity projection (MIP) of T1-weighted MRI scans were coregistered with segmented PET 3D regions of interest (ROIs) to annotate tumors (right). (**E**) Tumor-to-normal brain ratios calculated from maximum standardized uptake value (SUV_max_) for ^11^C nicotinamide in tumor versus contralateral brain. Mean ± SD, Veh: *n*_mice_ = 4, Dex: *n*_mice_ = 6. (**F**) Experimental design for combined methionine-restricted diet and Dex treatment in T16 tumor–bearing mice. (**G**) Body weights of individual mice treated as in (F). (**H**) Serum methionine levels in mice treated as in (F). Mean ± SD, *n*_mice_ = 5. (**I**) Tumor volumes from MRI of mice treated as in (F). Mean ± SD, *n*_mice_ = 5, except “Dex -, Met 100%” *n*_mice_ = 4. (**J** and **K**) Ki67 IHC of brain sections from mice treated as in (F). (J) Right shows higher magnifications of boxed regions. (K) Quantification of Ki67-positive tumor cells. Mean ± SD, *n*_mice_ = 5. (**L**) Methionine levels in tumor, contralateral brain, and liver tissues from mice treated as in (F). Mean ± SD, *n*_mice_ = 5. (**M**) Graphical summary: Dex boosts the tumor-specific NNMT expression converting nicotinamide to *N*^1^-methylnicotinamide. The positive charge of *N*^1^-methylnicotinamide favors its retention by glioblastoma cells enabling tumor PET imaging with nicotinamide tracers. Dex-dependent rewiring of methionine metabolism sensitizes glioblastoma to methionine restriction diet. *P* values were calculated using two-tailed, homoscedastic Student’s *t* tests: ratio paired (C) and unpaired [(E) and (H) to (L)].

The irreversible NNMT-dependent *N^1^*-methylation of nicotinamide introduces a positive charge on its pyridine ring, suggesting a possible intracellular trapping (Fig. 3D). Transport of *N*^1^-methylnicotinamide in the extracellular environment has been shown to depend on yet to be identified organic cation carriers (*32*). On these bases, we hypothesized that the augmented activity of NNMT selectively found in glioblastoma tumors (Fig. 4, I, R, and T) could be exploited for tumor metabolic imaging. To test this hypothesis, we administered radiolabeled ^14^C-nicotinamide to glioblastoma-bearing mice and performed autoradiography of brain microsections (Fig. 5A). The resulting autoradiography images delineate areas exposed to higher radioactivity corresponding to the tumor as detected by MRI and by subsequent histologic staining (Fig. 5B). The autoradiography signal in tumor areas was 1.9-fold higher than in the normal contralateral brain (Fig. 5C).

These encouraging results prompted the development of a positron-emitting ^11^C-nicotinamide tracer for live PET imaging. PET images acquired with this tracer in tumor-bearing mice show enhanced signal in brain areas defined as tumors by MRI (Fig. 5D). As demonstrated with stable isotope tracing, uptake of nicotinamide (Fig. 4, O and P) and its conversion to NAD^+^ are comparable between the tumor and the contralateral brain (Fig. 4Q), suggesting that the increase in ^11^C-nicotinamide-derived signal observed in the tumor (Fig. 5, D and E) is consistent with the accumulation of *N*^1^-methylnicotinamide (Fig. 4R). Consistent with the tumor-selective increase in NNMT expression and *N*^1^-methylnicotinamide accumulation (Fig. 4, I, J, and R), the tumor–to–contralateral brain ratio of the ^11^C-nicotinamide PET signal was increased in dexamethasone-treated mice (Fig. 5E). Overall, these results constitute preclinical proof of principle to develop nicotinamide-based PET tracers for the detection and metabolic visualization of glioblastoma in the clinical setting.

Next, we moved to test in vivo the therapeutic strategy indicated by our cell-based findings. The results obtained with glioblastoma spheroids (Fig. 3Q) suggested that the decreased methylation capacity reached by combining dexamethasone with methionine restriction could limit glioblastoma growth. To evaluate the antitumoral effects of this approach, mice with advanced glioblastoma tumors were exposed to a diet containing 5% of methionine compared to the control diet (Fig. 5F). The combination of this diet with dexamethasone did not cause greater body weight loss than each individual treatment (Fig. 5G). Unexpectedly, the methionine-restricted diet alone did not lower methionine levels in blood circulation, but it normalized the significant increase in methionine caused by dexamethasone treatment (Fig. 5H). MRI analysis after 3 weeks of treatment showed a 28% decrease in tumor volume in mice exposed to the combination treatment compared to vehicle-treated mice on control diet, although this difference did not reach statistical significance (Fig. 5I). Consistent with results obtained in 3D culture of T16 cells (Fig. 1C), dexamethasone significantly decreased the percentage of Ki67^+^ cells in tumors compared to vehicle-treated controls (Fig. 5, J and K). The methionine-restricted diet alone did not affect the proportion of proliferating Ki67^+^ glioblastoma cells compared to mice on control diet, but it significantly decreased this proliferative index by 35% when combined with dexamethasone (Fig. 5, J and K).

In line with the lack of apparent systemic methionine depletion observed upon methionine restriction diet (Fig. 5H), methionine levels were not decreased in tumor, contralateral brain, and liver tissue (Fig. 5L). A limitation of these measurements is that serum and tissue samples were not collected at a defined interval after synchronized feeding, which could have missed transient differences in circulating methionine caused by the diet. However, methionine levels were significantly decreased in brain tissue by 49% and in tumor tissue by 56% selectively in mice undergoing the combination treatment (Fig. 5L). These results show that dexamethasone treatment rewires methionine metabolism systemically and, when coupled with a methionine restriction diet, results in a brain-specific shortage of methionine that limits the growth of glioblastoma tumors (Fig. 5M).

## DISCUSSION

For half a century, patients with glioma have been administered synthetic glucocorticoids to manage the dismal effects that tumor edema has on brain function, quality of life, and life expectancy. In this study, we show that alongside its anti-inflammatory (*33*) and immunomodulatory (*34*) actions, dexamethasone has direct effects on glioblastoma metabolism that can be exploited to expand the diagnostic and therapeutic options for patients with glioblastoma.

In a panel of heterogeneous naïve human glioblastoma cell lines cultured in conditions that promote expression of neural stem cell markers (Fig. 1E), we identified a transcriptional signature induced by clinically relevant concentrations of dexamethasone (Fig. 1, G to H). Among the genes consistently up-regulated by dexamethasone, two glutamate transporters (fig. S2C) contributed to shift glioblastoma cells toward an astrocytic-like state (Fig. 2A and fig. S2, A and B), metabolically characterized by avid uptake of glutamate from the extracellular environment (Fig. 2, C and D), and its conversion into glutamine via glutamine synthetase (Fig. 2, E and F) (*2*). As a result, when cultured in physiological conditions, naïve glioblastoma cells do not substantially depend on glutamine uptake for anabolic processes as previously reported (Fig. 2D) (*35*).

Integration of transcriptomics and untargeted metabolomics identified NNMT-dependent production of *N*^1^-methylnicotinamide as the most prominent metabolic reaction positively regulated by dexamethasone in all glioblastoma cell lines tested (Fig. 3, A to M). Dexamethasone causes discordant effects on proliferation of different glioblastoma lines (Fig. 1A) and, even in the same cell line, has differential effects on growth depending on culture conditions (compare Fig. 1A with Fig. 1B). Nonetheless, it consistently promoted *N*^1^-methylnicotinamide synthesis across cell lines and culture conditions (Fig. 3, G to K), excluding a direct causal effect of NNMT on glioblastoma cell proliferation. The dexamethasone-dependent overexpression of NNMT is likely relevant to tumor types other than glioblastoma, as suggested by a proteomic screen that identified “nicotinamide metabolism” as a process activated by GR in breast cancer cells (*36*).

Our studies with spheroids (Fig. 1B) and brain xenografts indicate that dexamethasone slows down proliferation of glioblastoma cells (Fig. 4, E and G) and tumor growth (Fig. 4, C and D) through cancer cell-autonomous effects. We show that higher expression of *NNMT* mRNA in glioblastoma compared to nontumor samples (*37*) is amplified by dexamethasone that further stimulates expression of NNMT in glioblastoma cells (Fig. 4, J and K), contributing to the differential levels of *N*^1^-methylnicotinamide between the tumor and edematous brain tissue observed in patients with glioblastoma (Fig. 4T). The selective induction of NNMT in glioblastoma tumors but not in contralateral brain tissue (Fig. 4, I and J) is not due to differences in drug levels or GR expression between the tissues (Fig. 4, H and L) and may reflect tumor-specific factors, such as higher baseline NNMT expression (Fig. 4, K and T) (*37*), chromatin accessibility, or cofactor availability. Our results suggest that dexamethasone shifts glioblastoma cells toward a more therapy-resistant mesenchymal- astrocytic-like state (Fig. 2A and fig. S2, A and B) but do not support a causal role for NNMT in promoting glioblastoma progression (*38*).

In tumors of patients with glioblastoma, NNMT outcompetes NAMPT, the enzyme responsible for NAD^+^ production, for nicotinamide (Fig. 3S). This form of vitamin B3 efficiently permeates the brain (Figs. 3, R and S, and 4, O and P) where the tumor-specific methyltransferase activity of NNMT introduces a positive charge on its pyridine ring. These findings provided a clear mechanistic rationale to design a nicotinamide-based PET tracer for the metabolic imaging of glioblastoma, which can be enhanced by the administration of dexamethasone (Fig. 5E). Our approach shows the feasibility of radiochemical synthesis of ^11^C-nicotinamide, and the imaging results demonstrate its suitability to visualize tumors in orthotopic models of glioblastoma (Fig. 5D), strengthening the case for clinical development of novel PET tracers based on vitamin B3 analogs.

Last, we show that dexamethasone promotes the diversion of methionine-derived methyl groups toward *N*^1^-methylnicotinamide, sensitizing glioblastoma tumors to methionine restriction. Our results align with a recent report showing that methionine restriction diet as single treatment did not significantly decrease the levels of methionine and growth of another intracranial glioblastoma tumor model (*39*). Our working model, supported by evidence obtained in culture (Fig. 3, O to Q) and in vivo (Fig. 5, I to L), suggests that a methionine-restricted diet would synergize with the systemic rewiring of methionine metabolism imposed by dexamethasone, resulting in a brain-specific metabolic state with antitumor effects for glioblastoma (Fig. 5M). Given the scarcity of effective therapies for glioblastoma, we believe that these results achieved by combining dexamethasone, already a de facto standard of care for many patients with glioblastoma, with methionine restriction diet in a clinically relevant model represent a meaningful advance that warrants further exploration.

## MATERIALS AND METHODS

### Cell cultures

Glioblastoma cell lines were provided by S. Niclou, Luxembourg Institute of Health [P3, P13, and T16 were prepared as described in (*40*)], by H. Miletic, University of Bergen (BG7) (*41*), and by G. Morrison (Cancer Research UK Glioma Cellular Genetics Resource, E1, E15, E22, E25, E27, E30, E34, E55, and E56). All the cell lines were routinely cultured in minimum essential medium (MEM; Gibco, 21090-022) supplemented with Albumax II lipid-rich bovine serum albumin (BSA; 400 mg/liter; Gibco, 11021-037), human FGF-basic (20 ng/ml; PeproTech, AF-100-18B), human EGF (20 ng/ml; PeproTech, AF-100-15), heparin (2 μg/ml; Sigma-Aldrich, H3393), insulin-transferrin-selenium [(ITS); Gibco, 41400], 0.65 mM l-glutamine (Gibco, 25030-024), 0.1 mM non-essential amino acids (Gibco, 11140-035), 0.1 mM sodium pyruvate (Sigma-Aldrich, S8636), and vitamin B12 (0.0068 mg/liter; Sigma-Aldrich, V6629). All cell culture experiments were performed in Plasmax medium supplemented with the same concentration of Albumax II lipid-rich BSA, human FGF-basic, human EGF, heparin, and ITS as used in the MEM medium. The glioblastoma cells were routinely cultured as adherent monolayers for less than 15 passages on petri dishes coated with a thin layer of Matrigel (Corning, 356231). For coating, plates were incubated with a 2% Matrigel solution in Earle's balanced salt solution [(EBSS); Gibco, 24010-043] at 25°C for 1 hour. Matrigel solution in excess was removed, and cells were seeded.

All cell lines tested negative for mycoplasma infection using the MycoAlert Mycoplasma Detection Kit (Lonza, LT07-318). Short tandem repeat DNA analysis was performed on early cultures of BG7, P3, P13, and T16 cells.

### Cell proliferation assay

A total of 2 × 10^4^ to 6 × 10^4^ cells per well were seeded on 24-well plates coated with Matrigel. At the times indicated in figures, cells were quickly washed three times with phosphate-buffered saline (PBS), and 400 μl of 2.5% trypsin (Gibco, 15090-046) was added to each well. Cells were resuspended in PBS and counted with a CASY cell counter (Roche Applied Science).

### 3D spheroid cultures

A total of 5 × 10^2^ to 2 × 10^3^ cells per well were seeded in round-bottom ultralow attachment 96-well plates (Corning, 7007) in Plasmax medium. Twenty-four hours after seeding, the medium was changed and supplemented with the compounds or vehicle described in the figure legends. The medium was partially refreshed every 6 to 7 days to prevent nutrient exhaustion. Spheroid microphotographs were acquired every 6 to 7 days with a light microscope and analyzed with ImageJ software to quantify the area of the well covered by each spheroid.

### Confocal microscopy

Glioblastoma cells were plated on Matrigel-coated coverslips in 12-well plates and treated with vehicle or 1 μM dexamethasone for 72 hours. Cells were then gently washed three times with PBS before fixation using a 4% paraformaldehyde solution in PBS (pH 7.4) for 30 min and washing twice with ice-cold PBS (3 min). Cells were permeabilized for 3 min with a 0.1% Triton X-100 in PBS solution and then washed with PBS three times. Cells were then incubated for 30 min with a blocking solution of 1% BSA in PBS-Tween and then with anti-GR (1:50; Cell Signaling Technology, 12041S) and anti-Nestin (1:100; Abcam, ab134017) antibodies in 1% BSA in PBS-Tween in a humidified chamber for 1 hour at room temperature. Cells were then washed three times in PBS (5 min) and incubated with a solution containing two secondary antibodies raised in two different species and conjugated with two different fluorochromes in 1% BSA PBS solution for 1 hour at room temperature. Cells were washed with PBS three times in the dark, and nuclei were stained with 4′,6-diamidino-2-phenylindole. After three washes in PBS, coverslips were mounted with a drop of mounting medium, and the slides were stored in the dark at 4°C. Images were acquired on a Zeiss LSM 710 confocal microscope equipped with a Plan-Apochromat 63×/1.40 oil differential interference contrast (DIC) M27 objective using the following lasers 405, 488, and 561 nm and filters 410 to 462, 494 to 552, and 591 to 712 nm.

### RNA sequencing

A total of 3 × 10^5^ to 5 × 10^5^ cells per well were seeded in six-well plates coated with 2% Matrigel and cultured in Plasmax medium for 3 days. After 3 days, the medium was refreshed, and 24 hours later, the cells were counted using a CASY cell counter. The cells were detached with Accutase (StemPro, Gibco, A11105-01) and collected. RNA was extracted using the QIAshredder (QIAGEN, 79656) and the RNeasy Mini Kit (QIAGEN, 74104). On-column deoxynuclease-ribonuclease digestion was performed, and 1.5 μg of RNA was diluted in 50 μl of water to check the quality of the purified RNA. Agilent 2200 TapeStation (D1000 screentape) and Qubit (Thermo Fisher Scientific) were used to assess quality and quantity of the cDNA libraries prepared as described previously (*42*). The libraries were run on the Illumina NextSeq 500 using the High Output 75 cycles kit (2 × 36 cycles, paired-end reads, single index). A polyadenylated selection was performed to enrich for mRNAs. The Illumina sequence data were demultiplexed using bcl2fastq version 2.20.0.422 [bcl2fastq2 Conversion Software v2.20 Software Gudie (15051736)]. Quality checks on the raw RNA-seq data files were done using FastQC version 0.12.0 (https://www.bioinformatics.babraham.ac.uk/projects/fastqc/) and FastQ Screen version 0.15.2 (*43*). Additional quality checks and trimming were performed with FastP version 0.19.7 (*44*). RNA-seq paired-end reads were aligned to the 38.95 version of the human genome and annotation (*45*), using HiSat2 version 2.1.0 (*46*), and sorted using Samtools version 1.7 (*47*). Aligned genes were identified using HTSeq version 0.9.1 (*48*).

Expression levels were determined and statistically analyzed using the R environment version 3.5.3 (https://R-project.org/) and using packages from the Bioconductor data analysis suite (*49*). Differential gene expression was analyzed on the basis of the negative binomial distribution using the DESeq2 package version 1.22.2 (*50*) and adaptive shrinkage using Ashr (*51*). Computational analysis was documented at each stage using MultiQC (*52*), Jupyter Notebooks (*53*), and R Notebooks (https://rstudio.com/categories/integrated-development-environment/).

### Gene set enrichment analysis

Median ratio normalized read counts were used as an input for GSEA, performed using the Broad Institute software v4.2.3. Publicly available published signatures were used for GSEAs. Weighted signal to noise metric was used with 1000 gene set permutations. Normalized enrichment scores and false discovery rate significance values were obtained for each gene set.

### Harmonized dataset

Single-cell RNA-seq analysis was performed using a publicly available integrated and harmonized dataset from glioblastoma (*23*) on the CELLXGENE platform (https://cellxgene.cziscience.com). This dataset is composed of 16 integrated datasets, with 338,564 cells from a total of 110 patients. The dataset comprises 127,521 neoplastic cells and 211,043 cells from the local microenvironment, all annotated at multiple levels. The gene signature constituted by the 79 genes identified by RNA-seq and commonly up-regulated by dexamethasone in all four cell lines (BG7, P3, P13, and T16) was used as input to investigate this database focusing on neoplastic cells. High-expressing cells were selected, and their annotations were extracted as depicted in the ridgeline plots in Fig. 2A.

### Metabolite extraction

Adherent monolayers of glioblastoma cells were seeded in six-well plates coated with 2% Matrigel at 2 × 10^5^ (P3), 2.5 × 10^5^ (P13 and T16), 3 × 10^5^ (BG7), 3.5 × 10^5^ (E55, E56, and E27), 4 × 10^5^ (E22 and E27), 4.5 × 10^5^ (E1, E15 E21 E30, and E34) cells per well and cultured in Plasmax medium supplemented with vehicle (ethanol) or 0.1 μM dexamethasone. After 3 days, Plasmax medium was refreshed, and 24 hours later, the cells were extracted. The cells were quickly washed three times with ice-cold PBS, and the intracellular metabolites were extracted for 5 min at 4°C with 400 μl of ice-cold extraction solution [20% water, 30% acetonitrile (VWR, 75–08-5), and 50% methanol (VWR, 67-56-1)]. The extracted samples were centrifuged for 10 min at 12,000*g* and 4°C to remove cellular debris. The clear supernatant was transferred to liquid chromatography–mass spectrometry (LC-MS) glass vials and stored at −74°C until analyzed. The six-well plates were air-dried and stored at 4°C until the protein amount was quantified with a modified Lowry protein assay (*54*).

3D spheroids were extracted as previously described (*42*). Briefly, glioblastoma cells were grown in ultralow attachment surface 75-cm^2^ flasks (Corning, 3814) to favor the aggregation of suspension spheroids. Cells were incubated in Plasmax medium supplemented with vehicle (ethanol) or 0.1 μM dexamethasone for 3 days after which the spheroids were transferred to a 50-ml falcon tube, centrifuged for 5 min at 300*g*, resuspended in 50 ml of freshly prepared Plasmax, and transferred back to the ultralow attachment flask. After 24 hours, the spheres were collected in 30 ml of medium, transferred to a 50-ml falcon tube, and spun for 1 min at 600*g*. The pellet was transferred to an Eppendorf tube, washed with 1 ml of ice-cold PBS, and spun at maximal speed for 10 s. The PBS was removed, and the spheres were extracted in 1 ml of extraction solution, vortexed, and incubated for 5 min at 4°C. The extracted samples were then centrifuged (10 min at 13,000*g*, 4°C), and the supernatants were transferred to LC-MS vials, whereas the cellular pellets were stored at −20°C for protein quantification with a modified Lowry protein assay. Briefly, a standard curve was prepared with BSA (0 to 4 mg) in 50-ml falcon tubes. Seven hundred fifty microliters of 0.5% sodium deoxycholate in 1 M NaOH was added to the frozen pellets obtained upon metabolic extraction of the spheroids. The pellets were vortexed and sonicated with 2 to 3 cycles of sonication. The solution was transferred to 50-ml falcon tubes and shaken for 30 min. A total of 7.5 ml of a solution containing sodium copper EDTA (0.25 g/liter), Na_2_CO_3_ (20 g/liter), and NaOH (4 g/liter) was added to each sample, and the tubes were vigorously shaken for 10 min. Seven hundred fifty microliters of Folin and Ciocalteu’s phenol reagent (Merck Sigma-Aldrich, F9252) were added, and the tubes were shaken slowly for 30 min. Two hundred microliters per sample was transferred to a 96-well plate, and the absorbance was read at 680 nm in a plate spectrophotometer.

For mouse-derived tissues, frozen tissue fragments (10 to 30 mg) were weighted and extracted in 25 μl of extraction solution/mg of tissue. The tissue samples were homogenized by using the Precellys Lysing Kit CK14 (VWR, 10144-554). For the serum samples, 5 μl was diluted in 245 μl of extraction solution. The extracted tissues and sera were centrifuged for 10 min at 12,000*g*, 4°C, and the supernatants were stored at −80°C until LC-MS analysis.

### Targeted and untargeted metabolic analysis

The LC-MS was performed as described in (*55*). A Q Exactive Plus Orbitrap Mass Spectrometer (Thermo Fisher Scientific) was used coupled with an Ultimate 3000 high-performance liquid chromatography (HPLC) system (Thermo Fisher Scientific). The monolayer-, spheroid-, or tissue-extracted samples were injected (5 μl) and separated on a ZIC-pHILIC column (SeQuant; 150 mm by 2.1 mm, 5 μm; Merck KGaA, Darmstadt, Germany) coupled with a ZIC-pHILIC guard column (SeQuant; 20 mm by 2.1 mm) held at 45°C. The chromatographic separation was performed with a resolution of 35,000 [at 200 mass/charge ratio (*m/z*)] with electrospray ionization and polarity switching, to detect both positive and negative ions over a mass range of 75 to 1000 *m/z*. The metabolites were separated with a 15-min mobile phase gradient, which started at 80% acetonitrile/20% ammonium carbonate (20 mM, pH 9.2) and decreased to 20% acetonitrile/80% ammonium carbonate with a flow rate of 200 μl/min (total run time of 24.5 min). Compound Discoverer software (version 2.1.0.401, Thermo Fisher Scientific) was used to perform the untargeted metabolomic analysis. The retention times were aligned across all samples [maximum shift, 2 min; mass tolerance, 5 parts per million (ppm)]. The unknown compounds were detected and grouped using a minimum peak intensity of 1 × 10^5^, a mass tolerance of 5 ppm, and a maximum peak width of 0.5 min (retention time tolerance, 0.2 min). The compound Discoverer “Fill Gap” feature was used to fill the missing values (mass tolerance, 5 ppm; signal/noise tolerance, 1.5). The compounds were identified by comparing the mass and retention time of experimental peaks to an in-house library generated using commercial standards. Data-dependent fragmentation was also used to aid metabolite identification by matching the MS2 fragmentation spectra to mzCloud (https://mzcloud.org/). LC-MS analysis was carried out as described above using a representative sample, and the Q Exactive was operated in positive and negative polarity mode separately, with the addition of a data-dependent fragmentation experiment (MS2, top 10, min automatic gain control target 1 × 10^3^, max injection time = 100 ms, normalzed colliision energy: 25, 60, and 95). TraceFinder software (version 4.1, Thermo Fisher Scientific) was used to perform the targeted metabolomic analysis. Metabolite peak areas were determined by using the exact mass of the singly charged ions (5 ppm, mass accuracy), and the RT from an in-house library was generated using commercial standards (5 ppm, mass accuracy) analyzed through the same LC-MS apparatus. For both the untargeted and targeted metabolomic analyses, the peak areas were normalized for the micrograms of protein per well determined with a modified Lowry assay, or the total extracted microliters of serum, or the milligrams of tissue.

### Cell-media exchange rates

The rate of consumption and secretion of nutrients and metabolites from and to the cell culture medium were calculated as described in (*56*). Briefly, the extracellular medium was diluted 1:50 in extraction solution. The medium extracted from cell-free wells was used as reference. Media extracts were analyzed by LC-MS as described above. Aliquots of freshly prepared Plasmax medium with nutrients diluted at different concentrations (0.25×, 0.5×, 1×, 2×, and 4×) and spiked with higher concentrations of lactate were processed and used for metabolite quantification. For each metabolite (*x*), the exchange rate per day was quantified using the following equation

$$x=\frac{\left(\text{nmol in spent medium}-\text{nmol in cellfree medium}\right)}{\left(\mu g\;\text{prot}\;\text{day}\;0+\mu g\;\text{prot}\;\text{day}\;2\right)/2}$$

For the determination of glutamine and glutamate concentrations reported in Fig. 2D, media samples were transferred to a 96-well plate. The plate was sealed and loaded onto the YSI 2950 Biochemistry Analyzer (Xylem Inc.) and processed as per the manufacturer’s instructions.

### Immunoblotting

Cells were washed twice with ice-cold PBS, and proteins were extracted with radioimmunoprecipitation assay (RIPA) buffer (EMD-Millipore, 20-188) containing Pierce protease and phosphatase inhibitors (Thermo Fisher Scientific, A32961). Protein amounts were quantified with a standard bicinchoninic acid assay (Pierce, A32961), while mouse tissues were extracted with 25 μl of RIPA buffer per mg of wet weight. Mouse tissue fragments were homogenized with the Precellys Lysing Kit. Forty to 80 μg of protein extract was loaded in 9.5% acrylamide gels for electrophoresis and blotted onto nitrocellulose membranes. PageRuler Prestained Protein Ladder (Thermo Fisher Scientific, 26616) was used as a reference for the proteins’ molecular weight. The membranes were incubated overnight with the following primary antibodies: CD133 (1:1000; Abcam, ab226355), GR (D6H2L) (1:1000; Cell Signaling Technology, 12041S), glutamine synthetase (1:1000; BD Biosciences, 610517), Nestin (1:1000; Abcam, ab134017), NNMT (1:500; Abcam, ab119758), Sox2 (1:1000; Abcam, ab97959), UGDH (1:1000; Abcam, ab246999), and vinculin (1:1000; Cell Signaling Technology, 13901). Secondary antibodies [anti-mouse IRDye 800CW (LI-COR, 926-32212) and anti-rabbit IRDye 680RD (LI-COR, 926-68073)] were used, and the gels were visualized with an Odyssey infrared scanner (LI-COR).

### siRNA transfection

T16 glioblastoma cells were transfected with small interfering RNAs (siRNAs) using DharmaFECT transfection reagents (Horizon Discovery, T-2002-02) according to the manufacturer’s instructions, with adaptations for the six-well plate format. Shortly, cells were seeded at 5 × 10^5^ cells per well in antibiotic-free complete medium and incubated overnight at 37°C with 5% CO_2_. For complex formation, siRNAs were diluted in serum-free Plasmax medium to a volume of 200 μl (final concentration, 2.5 nM). Separately, DharmaFECT reagent was diluted in serum-free medium (1.25 μl of reagent in 198.75 μl of medium). Both solutions were incubated for 5 min at room temperature before mixing. The combined solution (400 μl) was incubated for 20 min at room temperature and then supplemented with 1.6 ml of antibiotic-free complete Plasmax medium to yield a final transfection volume of 2 ml. Culture medium was aspirated from six-well plates, and 2 ml of transfection medium was added per well. The transfection medium was replaced with fresh complete Plasmax medium after 24 hours to minimize cytotoxicity. Cells were incubated at 37°C with 5% CO_2_ for 96 hours and for an additional 48 hours in the presence of 0.1 μM dexamethasone (Sigma-Aldrich, D4902) or the corresponding volume of ethanol (vehicle control). Each transfection experiment included nontargeting negative control siRNA (Horizon Discovery, D-001810-01-10. siRNA1: UGGUUUACAUGUCGACUAA; siRNA2: UGGUUUACAUGUUGUGUGA; siRNA3: UGGUUUACAUGUUUUCUGA; siRNA4: UGGUUUACAUGUUUUCCUA) alongside the GR siRNA (NR3C1: L-003424-00. siRNA1: GAACUUCCCUGGUCGAACA; siRNA2: GGAAACAGACUUAAAGCUU; siRNA3: UGACAAAACUCUUGGAUUC; siRNA4: GCAUGUACGACCAAUGUAA).

### In vivo studies

Animal experiments were either approved by the animal welfare structure of LIH and the national authorities responsible for animal experiments in Luxembourg under the reference LRNO-2016-01 or performed in accordance with UK Home Office Regulations (project licenses 70/8645 60/4181, PP6345023, and PP0604995) and subject to review by the Animal Welfare and Ethical Review Board of the University of Glasgow. Except for the pharmacokinetic experiments in Fig. 4 (A and B), in-house bred nonobese diabetic severe combined immunodeficient gamma (NSG) female mice were used and housed in ventilated cages with ad libitum food and water access and 12-hour light/12-hour dark cycles. The temperature of the facility was kept between 19° and 23°C with 55 ± 10% humidity.

To assess the pharmacokinetics of dexamethasone, in-house bred NSG male and female mice 18 to 22 weeks of age were injected intraperitoneally with the clinically relevant dose of dexamethasone-21-phosphate (2 mg/kg) (*57*). This water-soluble prodrug is rapidly dephosphorylated by intracellular esterases to release dexamethasone (*26*). NSG mice were euthanized 1, 3, 6, 12, and 24 hours after the dexamethasone administration, and serum and brain tissues were collected for LC-MS analysis.

### Dexamethasone effect on the growth of orthotopically implanted glioblastoma tumors

For the experiment reported in Fig. 4 (C to L), Swiss nude female mice 8 to 12 weeks of age were orthotopically implanted with T16 tumor organoids at the Norlux Neuro-Oncology Laboratory of the LIH (*58*). For all the other in vivo experiments, T16 tumor organoids were injected orthotopically in NSG mice 6 to 22 weeks of age at the Cancer Research UK Scotland Institute, as described in (*59*). Mice were allocated to treatments to obtain aged-matched groups. Tumor formation was assessed by T2 MRI, and tumor-bearing mice were randomized to vehicle (0.9% NaCl solution) or dexamethasone-21-phosphate disodium salt (2 mg/kg) administered by intraperitoneal injection 5 days/week for the experiment performed at the LIH or 7 days/week for the experiments performed at the Cancer Research UK Scotland Institute. Tumor volumes were monitored by T2 MRI, and mice reaching the experimental endpoint were euthanized 4 hours after the last injection. Tissue and serum samples were collected for metabolomic analyses (described above) or fixed in paraformaldehyde (4%) solution for immunohistochemistry (IHC).

### Nicotinamide tracing in mice

NSG mice were transplanted with T16 organoids, and 6 weeks after transplantation, they were treated with vehicle or dexamethasone-21-phosphate (Dex) as described above. Three and a half hours after the last Dex administration and 30 min before sacrifice, mice were injected intraperitoneally with ^15^N-nicotinamide [nicotinamide-(amide-^15^N), 592021, Merck] dissolved in 0.9% NaCl solution at the dose of 11.5 μl/g of body weight and 37 mg/kg. After euthanasia, the brain was dissected, and tissue fragments of the tumor and contralateral hemisphere were snap-frozen in dry ice and stored at −74°C until processed for metabolic analysis.

### Methionine restriction diet

NSG mice were transplanted with T16 organoids, and 6 to 7 weeks after transplantation, the mice were randomized, and their diet was switched from the normal chow to isocaloric diets (15.6 MJ/kg) containing 0.525% of methionine (control, S9074-E430, Ssniff Spezialdiäten GmbH) and 0.026% methionine (95% restricted methionine diet, S9074-E405, Ssniff Spezialdiäten GmbH). From the following day, the mice were treated daily with vehicle or dexamethasone-21-phosphate (Dex) by intraperitoneal injection. Mouse weights were recorded three times/week to assess the effect of the diet and the combination treatment. Tumor volumes were monitored by T2 MRI. Mice were fed ad libitum, and access to food was not time restricted. Mice reaching the experimental endpoint were euthanized 4 hours after the last injection. At this time point, tissue and serum samples were collected for metabolomic analyses (described above) or fixed in paraformaldehyde (4%) solution for IHC.

### Magnetic resonance imaging

For the mice intracranially implanted at the LIH, tumor growth was monitored by MRI as described in (*58*). Briefly, 2.5% isoflurane was used to keep the mice under anesthesia during the image acquisition, with breathing and temperature under constant monitoring. For routine follow-up, an MRI (Fast Spin Echo, FSE-T2 sequence, 3T MRI system, MR Solutions) was used, and a Fast Spin Echo T2-weighted MRI sequence was applied [field of view = 25 mm, matrix size = 256 by 248, echo time (TE) = 68 ms, repetition time (TR) = 3000 ms, and slice thickness = 1 mm]. MRI data were analyzed by ImageJ, and tumor volume was calculated by tumor delineation in each slide, multiplied by slice thickness.

For the MRI of mice implanted at the Beatson Institute, mice with intracranial xenografts were maintained under inhaled isoflurane (induction: 5%, v/v; maintenance: 1.5 to 2.0%, v/v) in ~95% oxygen during the entire imaging procedure. Whole brain T2 FSE 3D axial MRI scans (slice thickness, 1.0 mm; TR, 2000 ms; TE, 83.7 ms; flip angle, 90°) were performed using a preclinical nanoScan 1 T MRI scanner (Mediso Medical Imaging Systems, Hungary). For the quantitative assessments of tumor volumes, manual segmentation was performed blindly using VivoQuant ver4.0 (Invicro, USA).

### Immunohistochemistry

All hematoxylin and eosin (H&E) and IHC stainings were performed on 4-μm formalin-fixed paraffin-embedded (FFPE) sections previously kept at 60°C for 2 hours. The following antibodies were stained on an Agilent Autostainer: GR (Cell Signaling Technology, 12401) and Nestin (Abcam, ab6320). FFPE sections were loaded into an Agilent pretreatment module to be dewaxed and undergo heat-induced epitope retrieval (HIER) using either low or high pH target retrieval solution (TRS) (Agilent, K8005 and K8004). Sections for Nestin staining underwent antigen retrieval using low pH TRS, and sections for GR staining underwent antigen retrieval using high pH TRS. All sections were heated to 97°C for 20 min in the appropriate TRS. After HIER, all sections were rinsed in flex wash buffer (Agilent, K8007) before being loaded onto the autostainer. The sections underwent peroxidase blocking (Agilent, S2023) for 5 min and then were washed with FLEX wash buffer (Agilent, K8007). Sections for Nestin and GR were blocked using a mouse-on-mouse kit (Vector Lab, MKB2213-1) before primary antibody application for 35 min at a previously optimized dilution (GR, 1:2000; Nestin, 1:750). The sections were then washed with FLEX wash buffer before the application of appropriate secondary antibody for 30 min. Sections for Nestin had mouse envision applied (K4001, Agilent) and sections for GR staining had rabbit envision (K4003, Agilent) applied for 30 min. Sections were rinsed with FLEX wash buffer before applying liquid DAB (Agilent, K3468) for 10 min. The sections were then washed in water and counterstained with hematoxylin z (CellPath, RBA-4201-001). The sections for Ki67 (Cell Signaling Technology, 12202) staining were performed on a Leica Bond Rx autostainer. The FFPE sections underwent on-board dewaxing (Leica, AR9222) and antigen retrieval using ER2 solution (Leica, AR9640) for 20 min at 100°C. Sections were rinsed with Leica wash buffer (Leica, AR9590) before peroxidase block was performed using an Intense R kit (Leica, DS9263) for 5 min. Sections were rinsed with wash buffer before application of Ki67 antibody at 1:1000 dilution for 30 min. The sections were rinsed with wash buffer and incubated with rabbit envision secondary antibody for 30 min. The sections were rinsed with wash buffer, visualized using DAB, and then counterstained with hematoxylin in the Intense R kit. H&E staining was performed on a Leica autostainer (ST5020). Sections were dewaxed, taken through graded alcohols, and then stained with hematoxylin z (CellPath, RBA-4201-00A) for 13 min. Sections were washed in water, differentiated in 1% acid alcohol, and washed, and the nuclei blued in Scotts tap water substitute (in-house). After washing, sections were placed in Putt’s eosin (in-house) for 3 min. To complete H&E and IHC staining, sections were rinsed in tap water, dehydrated through graded ethanol, and placed in xylene. The stained sections were cover-slipped in xylene using DPX mountant (CellPath, SEA-1300-00A). HALO software was used to quantify the fraction of Ki67- and GR-positive nuclei in the tumor area.

### Brain autoradiography

For the autoradiography study, [carbonyl-^14^C]nicotinamide formulated in ethanol (ARC 0794 50 μCi, American Radiolabeled Chemicals Inc., USA) was dried and resuspended in 0.9% NaCl solution. Two hundred microliters of 150 kBq [carbonyl-^14^C]nicotinamide was injected per mouse as a bolus in the tail vein of NSG female mice bearing T16 brain tumors. Mice were anaesthetized with 2 to 2.5% isoflurane/95% O_2_ and maintained on a heated bed at 40°C for 5 min after the injection. The mice were then recovered from the anesthesia and euthanized 90 min postinjection. The brain was immediately collected, snap-frozen in isopentane (Merck Sigma-Aldrich), and stored at −74°C. Frozen brain sections (10 μm) were cut at −18°C with the Leica CM3050 S research cryostat (Leica Biosystems) and thaw-mounted onto Superfrost Plus glass slides (Thermo Fisher Scientific). The sections were air-dried and placed in an exposure cassette where the radioactivity was recorded for 30 days with a phosphor screen (Fuji BAS-IP SR, 20 cm–by–25 cm super-resolution screen, GE Healthcare, USA). Images of the exposed phosphor screen were acquired with a GE Typhoon FLA 7000 Biomolecular Imager (GE Healthcare) at a pixel size of 25 μm using a 650-nm laser beam and IP (BP390) filter for phosphorimaging. The same sections were manually stained with a standard H&E protocol and mounted with coverslip. Image analysis of autoradiograms was performed with ImageJ/Fiji (version 1.5x, National Institutes of Health, USA). The mean intensity of the regions of interest (ROIs) was corrected to the background levels adjacent to the brain areas.

### Radiosynthesis of 1-[^11^C]nicotinamide

Ultrapure nitrogen containing 0.1% oxygen, used as a target gas mixture, was purchased from BOC UK, and all the other reagents and materials were purchased from Merck Life Science UK. No-carrier-added [^11^C]carbon dioxide (CO_2_) was produced via the ^14^N(p, α)^11^C nuclear reaction by irradiation of an aluminum target filled with 99.9% N_2_/0.1% O_2_ with a 16.4-MeV proton beam on the PETtrace cyclotron (GE Healthcare, USA) at the Radiopharmaceutical Unit of the West of Scotland Glasgow PET Centre, Gartnavel Hospital, Glasgow. Typically, 105 GBq of [^11^C]CO_2_ at the end of bombardment (EOB) was obtained after 50-min target irradiation, with the current set at 44 μA. [^11^C]hydrogen cyanide (HCN) was produced on a customized SYNTHRA synthesizer (Synthra GmbH, Germany) by the standard procedure of converting [^11^C]CO_2_ into [^11^C]methane on a Pd catalyst with consequent conversion into [^11^C]HCN by reaction with ammonia gas over a Pt catalyst at 950°C as described previously (*60*, *61*). [^11^C]nicotinamide was synthesized by a one-pot radiolabeling method (*61*) which was adopted on the SYNTHRA module with some modifications. Briefly, the gaseous [^11^C]HCN, carried in the gas mixture of ammonia (5 ml/min) and helium (10 ml/min; total gas flow rate was kept at 15 ml/min), was trapped in the reaction mixture containing 2 mg of tetrakis(triphenylphosphine)Pd(0), 2 mg of Kryptofix222, 8 μl of 3-bromopyridine, 20 mg of sodium percarbonate, and 1 μl of 8 M KOH solution in 500 μl of anhydrous tetrahydrofuran at −10°C. The cyanation reaction was carried out for 2 min at 90°C. To improve the conversion of the intermediate [cyano-^11^C]cyanopyridine into [carbonyl-^11^C]nicotinamide, the potassium percarbonate reagent (50 mg) was predissolved in 1 ml of water and added to the reaction mixture after [^11^C]cyanation of 3-bromopyridine. The product purification and formulation were accomplished by semipreparative HPLC on the Synergi Hydro RP, 10-μm, 250 mm–by–10 mm column (Phenomenex, USA) with the 100 mM phosphate buffer (pH 6.8) containing 6% ethanol (v/v) mobile phase solvent. Peak fraction of 1-[^11^C]nicotinamide was collected at the retention time of 9 min and used in the imaging experiments. Total synthesis time was around 30 to 35 min after EOB. Overall, radiochemical yield was 15 ± 5%, volumic activity was 50 ± 10 MBq/ml, molar activity was 7 ± 2 GBq/μmol at injection time, and radiochemical purity was >99%.

### PET/MRI imaging

Ten female NSG mice at 12 to 16 weeks of age were implanted with T16 glioblastoma organoids. Starting at 22 days after implantation, the mice were administered 0.9% NaCl solution (vehicle control) or dexamethasone-21-phosphate disodium salt (2 mg/kg) by intraperitoneal injection 7 days/week. Eleven days after the treatment start, the mice were imaged on a nanoScan PET/MRI scanner (Mediso Medical Imaging Systems, Hungary), and the system was operated by Nucline NanoScan software (version 3.00, Mediso Medical Imaging Systems, Hungary). Mice were maintained on a double-bed heated at 37°C and kept under inhaled isoflurane anesthesia (induction: 5%, v/v; maintenance set at 1.5 to 2.0%, v/v) in 95% oxygen during injection and throughout the imaging procedure. A bolus of ~40 MBq of 1- [^11^C]nicotinamide per mouse was injected intravenously into the tail vein, and a PET scan was acquired from 20- to 70-min postinjection in the list mode. Sequentially, a T1-weighted 3D gradient echo (GRE) coronal/sagittal MRI sequence (slice thickness, 0.7 mm; 8 excitations; TR, 22.5 ms; TE, 3.8 ms; and 30° flip angle) was used to acquire whole-body MR images. The brain T2-weighted 3D FSE axial MRI sequence (slice thickness, 1 mm; 8 excitations; TR, 2000 ms; TE, 83.7 ms; and echo train length, 16) was used to acquire the brain MR images. 1T rat coil with a double mouse bed was used for the PET/MRI study. PET raw data were reconstructed with Nucline NanoScan software using the Tera-Tomo 3D reconstruction method in the dynamic reconstruction protocol, normal reconstruction resolution with reconstruction parameters of four iterations, six subsets, and the body-air-threshold value of 35%, between 20- to 40-min and 50- to 70-min postinjection (two 20-min bins). The binning parameters were set for the coincidence mode at 1 to 5 and the energy window at 400 to 600 keV, producing an isotropic matrix with a 0.4-mm voxel size. PET images were corrected for random coincidence events, attenuation, scatter, radioactivity decay, and dead time. The material map was generated from the whole-body T1-weighted GRE 3D MRI images. PET/MRI images were coregistered and analyzed by VivoQuant software (version 4.0, Invicro, USA). The reconstructed PET images were coregistered with the MRI images for anatomical reference using the automated method and manually refined in VivoQuant. Volumic ROIs were manually drawn over the tumor using the 3D ROI tool to obtain PET quantitative data and to segment the tumor and normal brain regions to visualize the ROIs on MRI. All PET/MRI images were generated by VivoQuant software. A Gaussian isotropic filter of 0.4 was applied for PET image smoothing. For PET image quantification, the maximum of ROI activity concentration was used to calculate the maximum standardized uptake value (SUV_max_) based on following formula: ${\text{SUV}}_{\text{max}}=\frac{\text{ROI}\;\text{activity concentration}\;\left(\text{Bq}/\text{ml}\right)}{\text{mouse weight}\;\left(g\right)\times \text{injected activity}\;\left(\text{Bq}\right)}$ , and the tracer uptake was expressed as the tumor to contralateral brain ratio. Maximum intensity projection image of T2-weighted MRI showing the brain tumor was rendered by 3D ROI tool on PET.

### Human studies

Samples from patients with glioblastoma, who gave informed consent under the study REK 2010/130-2, were collected at the Department of Neurosurgery, Haukeland University Hospital, Norway as described in (*30*). The tissue samples were stereotactically harvested using a biopsy forceps under neuronavigational guidance. The samples were collected from representative areas of tumor core, periphery and adjacent edematous brain which were identified preoperatively (1 to 2 weeks before surgery) on contrast-enhanced T1 MRI volume uptake.

For the nicotinamide tracing, three patients with glioblastoma (female aged 58, male aged 55, and male aged 62) gave informed consent under the study REK# 578316, and samples were collected at the Department of Neurosurgery, Oslo University Hospital, Norway. The patients were administered 250 mg of ^15^N nicotinamide (Sigma-Aldrich, 592021) in drinking water immediately before undergoing surgery for tumor resection. Blood samples were obtained before and after the administration of ^15^N nicotinamide at the times indicated in Fig. 3R. All patients received steroids (methylprednisolone) in the week preceding surgery as standard therapy regimen. LC-MS data were analyzed with TraceFinder (version 4.1) or Skyline (version 22.2.0.351) software to assess the levels of selected metabolites.

### Statistical analysis

The independent experimental replicate numbers and the statistical tests used are described in the figure legends. The statistical analysis of the untargeted metabolomics and RNA-seq were performed with Compound Discoverer (version 2.1.0.401, Thermo Fisher Scientific) and htseq count (https://htseq.readthedocs.io/en/ release_0.10.0/), respectively. For the statistical analysis of tumor growth, the compare groups of growth curves permutation test https://bioinf.wehi.edu.au/software/compareCurves/ to assess the significant differences between growth curves was used. For all other tests, GraphPad Prism 9.0 or later versions were used.
