# Supplementary material for: Steroid-dependent metabolic rewiring reveals novel therapeutic and imaging approaches for glioblastoma
Source: Sci Adv. 2026 Jan 23;12(4):eadx6539. doi: 10.1126/sciadv.adx6539 (PMC12829581; doi:10.1126/sciadv.adx6539)
Supplement: Supplementary file 1 — Figs. S1 to S3 [file sciadv.adx6539_sm.pdf]

Science Advances
AAAS

Supplementary Materials for

Steroid-dependent metabolic rewiring reveals novel therapeutic and imaging approaches for glioblastoma

Maria Francesca Allega *et al.*

Corresponding author: Saverio Tardito, saverio.tardito@meduniwien.ac.at

Sci. Adv. **12**, eadx6539 (2026)
DOI: 10.1126/sciadv.adx6539

This PDF file includes:

Figs. S1 to S3

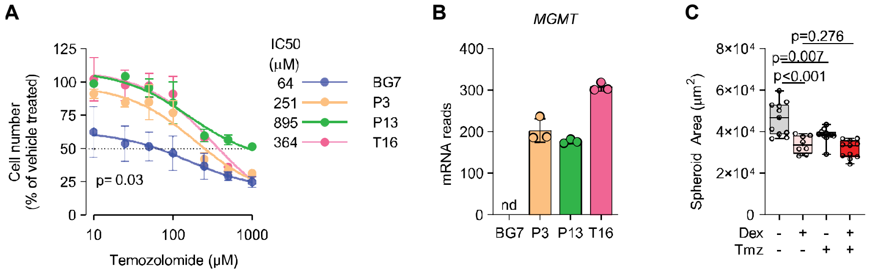

Fig. S1. Dexamethasone does not alter the growth response of *MGMT*-positive cells to temozolomide. (**A**) Cell number of glioblastoma cell lines cultured as monolayers and treated with the indicated concentrations of temozolomide (TMZ) for 4 days. The dose-response curves were obtained using a log(inhibitor) vs. response (three parameters) (GraphPad prism 10.2.3). P values refer to a two-tailed, homoscedastic Student's t test for paired samples comparing BG7 and P3 at 50 µM temozolomide. The values decreasing the relative number of cells to 50% of the control (IC50) are indicated. (**B**) Bar diagram of *MGMT* expression obtained by RNA sequencing. The *MGMT* mRNA was not detected (nd) in two out of three samples of BG7 cells that were therefore classified as *MGMT* negative. n_{exp}=3 as indicated by the data points. Bars represent mean ± s.d. (**C**) Proliferation assay of T16 glioblastoma cells cultured as spheroids treated with vehicle, 0.1 µM dexamethasone (Dex), and 250 µM TMZ as indicated. The area covered by each spheroid was quantified 20 days after cell seeding. Data points represent individual spheroids from two independent experiments. Boxes indicate the 25th-75th percentiles, lines the medians, and whiskers the minimum and maximum values. P values were calculated using a two-tailed, homoscedastic Student's t test for unpaired samples.

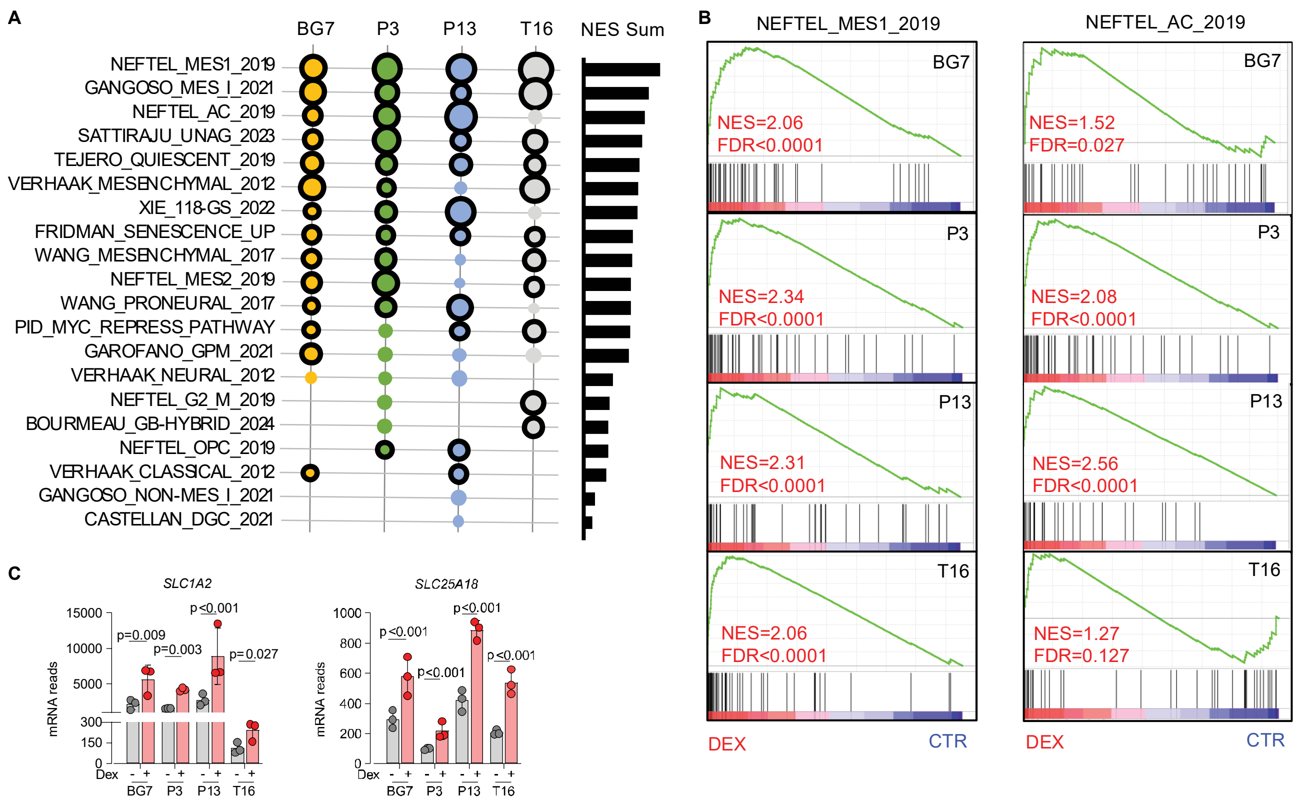

Fig. S2. Dexamethasone shifts glioblastoma cells towards a more astrocytic signature. (**A**) Bubble plot of GSEA analysis of BG7, P3, P13 and T16 cells for custom-published signatures. Size indicates normalized enrichment score (NES) and black borders mean FDR q values < 0.05. Pathways are ranked by the sum of NES in all cell lines, represented by the histograms. (**B**) GSEA plot of the MES1 and AC-like signatures from Neftel et al. (*62*) in BG7, P3, P13 and T16 cell lines after dexamethasone (Dex) treatment. NES and FDR q-value are indicated. (**C**) Expression levels of the *SLC1A2* and *SLC25A18* carriers as obtained by RNA sequencing. $n_{exp}=3$ as indicated by the data points. Bars represent mean ± s.d. P values were calculated with DESeq2 using a Benjamini-Hochberg method to correct for multiple testing.

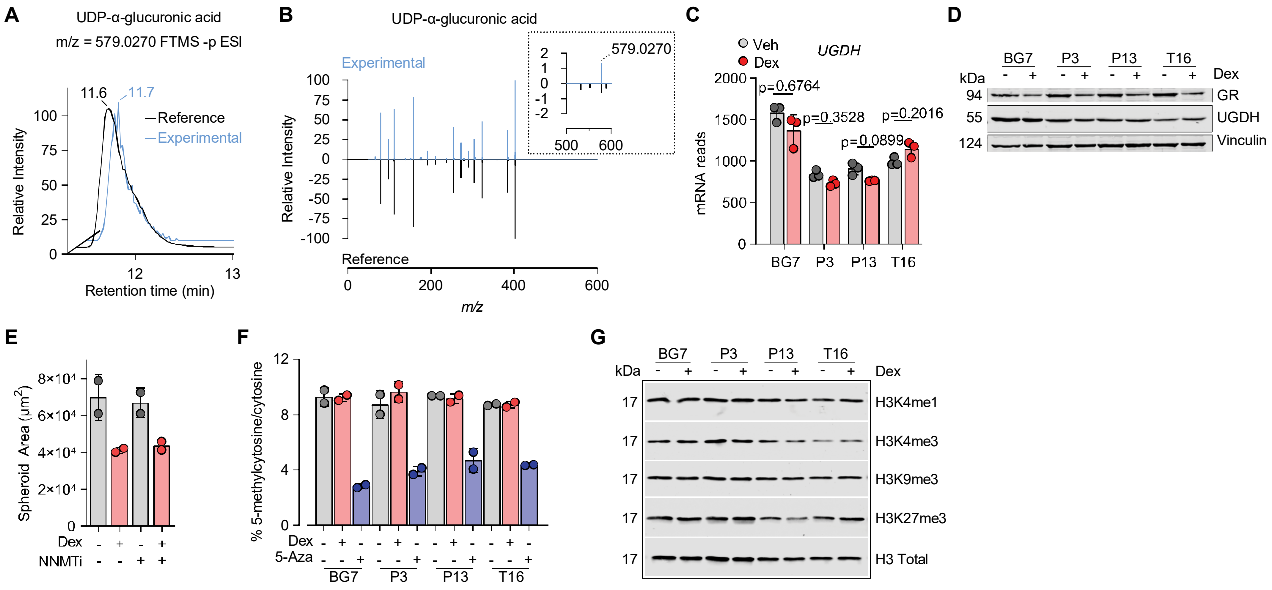

Fig. S3. Validation of Untargeted Metabolomics Hits and Functional Role of NNMT in Dexamethasone-Treated Cells. (**A**) Overlaid extracted ion chromatograms of m/z 579.0270 obtained from an intracellular extract of T16 cells and UDP-α-glucuronate chemical standard (reference). (**B**) Mirror plot of MS/MS spectra from an intracellular extract of T16 cells and UDP-α-glucuronate chemical standard (reference) comparing parent (m/z 579.0270) and fragment ions. The inset shows the spectra for low abundant ions with m/z between 500-600. (**C**) Expression levels of *UGDH* mRNA obtained by RNA sequencing. n_{exp}=3 as indicated by the data points. Bars represent mean ± s.d. P values were calculated with DESeq2 using a Benjamini-Hochberg method to correct for multiple testing. (**D**) Western blot analysis showing the expression of UGDH and GR in 4 glioblastoma cell lines cultured as monolayers and treated with vehicle or 0.1 µM dexamethasone (Dex) for 3 days. Vinculin is shown as a loading control. (**E**) Proliferation assays of T16 cells cultured as spheroids and incubated with or without 10 µM NNMTi (JBSNF-000088) for 27 days. The area covered by each spheroid was quantified from microphotographic images. n_{exp}=2 as indicated by the data points. Bars represent mean ± s.d. (**F**) Percentage of 5-methylcytosine over total cytosine quantified by LC-MS in extracted DNA of cells cultured for 6 days as adherent monolayers and incubated as in (G) and supplemented daily with 5 µM of the DNA methyltransferase inhibitor 5-azacytidine (5-Aza) as indicated. n_{exp}=2 as indicated by the data points. Bars represent mean ± s.d. (**G**) Western blot analysis showing the expression of different methylation marks of Histone 3 in 4 glioblastoma cell lines cultured as monolayers and treated with vehicle or Dex 0.1 µM for 3 days. Histone 3 Total is shown as a loading control.
